# Epithelial-Mesenchymal Transition in tumor microenvironment

**DOI:** 10.1186/2045-3701-1-29

**Published:** 2011-08-31

**Authors:** Yingying Jing, Zhipeng Han, Shanshan Zhang, Yan Liu, Lixin Wei

**Affiliations:** 1 Tumor Immunology and Gene Therapy Center, Eastern Hepatobiliary Surgery Hospital, the Second Military Medicial University, Shanghai, China

**Keywords:** Epithelial-Mesenchymal Transition (EMT), Tumor Microenvironment, Metastasis, Signaling pathway

## Abstract

The epithelial to mesenchymal transition (EMT) plays crucial roles in the formation of the body plan and also in the tumor invasion process. In addition, EMT also causes disruption of cell-cell adherence, loss of apico-basal polarity, matrix remodeling, increased motility and invasiveness in promoting tumor metastasis. The tumor microenvironment plays an important role in facilitating cancer metastasis and may induce the occurrence of EMT in tumor cells. A large number of inflammatory cells infiltrating the tumor site, as well as hypoxia existing in a large area of tumor, in addition many stem cells present in tumor microenvironment, such as cancer stem cells (CSCs), mesenchymal stem cells (MSCs), all of these may be the inducers of EMT in tumor cells. The signaling pathways involved in EMT are various, including TGF-β, NF-κB, Wnt, Notch, and others. In this review, we discuss the current knowledge about the role of the tumor microenvironment in EMT and the related signaling pathways as well as the interaction between them.

## Introduction

**Figure 1:**
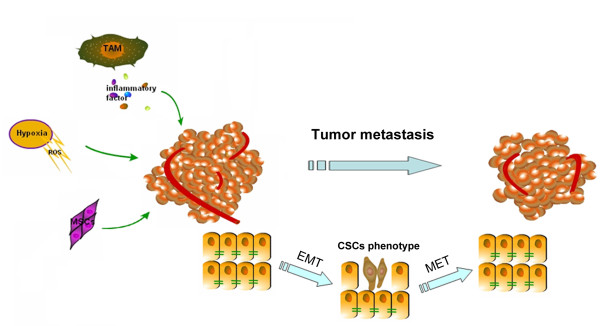
**Various factors that induce cancer cell Epithelial-Mesenchymal Transitions (EMT) in tumor microenvironment**. Inflammatory cells and cytokines, increase of reactive oxygen species (ROS) in mitochondria induced by hypoxia, mesenchymal stem cells all can effectively lead the epithelial-to-mesenchymal transition (EMT) of tumor cells. EMT is a key event in the tumor invasion process whereby epithelial cell layers lose polarity together with cell-cell contacts and then undergo a dramatic remodeling of the cytoskeleton. EMT also causes disruption of cell-cell adherence, loss of apico-basal polarity, matrix remodeling, increased motility and invasiveness in promoting tumor metastasis. Once migrating to the suitable site, tumor cells re-express E-cadherin and other epithelial markers via a process that is sometimes referred to as "mesenchymal-to-epithelial transition (MET)".

The main cause of death in patients is tumor progression with metastasis. Tumor metastasis arises from precursor lesions to the fully invasive, metastatic disease, which progress through histopathologically distinct stages, and epithelial-mesenchymal transition (EMT) is of potential importance for this process [1]. EMT plays crucial roles in the formation of the body plan and also contributes to tissue repair. EMT is also a key event in the tumor invasion process whereby epithelial cell layers lose polarity together with cell-cell contacts and then undergo a dramatic remodeling of the cytoskeleton [2]. In addition, EMT also causes disruption of cell-cell adherence, loss of apico-basal polarity, matrix remodeling, increased motility and invasiveness [3-5] in promoting tumor metastasis. Once migrating to the suitable site, tumor cells re-express E-cadherin and other epithelial markers via a process that is sometimes referred to as "mesenchymal-to-epithelial transition" (MET) (Figure 1) [6].

The tumor microenvironment is composed of inflammatory and immune cells, hypoxia, stromal, extracellular components including extracellular matrix (ECM), as well as soluble factors, and plays an important role in facilitating cancer progression and metastasis. Brabletz et al. [7] compared the central areas of primary colorectal cancer and corresponding metastases, and found that nuclear β-catenin was in dedifferentiated mesenchyme-like tumor cells at the invasive front and it was localized to the membrane and cytoplasm. This study suggested that the tumor microenvironment may induce the occurrence of EMT in tumor cells. A large number of inflammatory cells infiltrating the tumor, as well as hypoxia existing in a large area of tumor, in addition many stem cells present in tumor microenvironment, such as cancer stem cells (CSCs), mesenchymal stem cells (MSCs), all of these may be the inducers of EMT in tumor cells. Most recently, several intriguing studies have described the inducers of EMT and the underlying mechanisms. In this study, we summarize the main incentives for EMT in tumor microenvironment as well as the interaction between them.

### Inflammation as an Inducer of tumor EMT

The inflammatory component is an essential part of the malignant microenvironment [8]. Cordon-Cardo & Prives have established strong associations between chronic inflammatory conditions and tumourigenesis for decades [9]. Colon, gastric, liver and pancreatic carcinomas are all closely associated with ulcerative colitis, chronic gastritis, hepatitis and chronic pancreatitis respectively, which exemplify the close connection between inflammation and tumor appearance. Leukocyte infiltration, cytokines, and chemokines are crucial elements which contribute to cancer-related inflammation [10]. In addition to promoting carcinogenesis, tumor associated macrophages (TAMs) and their released factors (e.g. IL-1, TNF-α) have long been known to support all steps of invasion and metastasis [11,12]. An imaging study in vivo has shown that carcinoma cells migrate from mouse primary tumors through a process of EMT and that this process is dependent on an inflammatory microenvironment provided by the TAMs and other stromal cells such as the CAFs [13]. Recently, the new finding that TNF-α induces Snail promoter activity and EMT in MCF-7 breast cancer cells reinforced the connection between inflammation and EMT [14].

In addition to induce cancer EMT directly, TNF-α can up-regulate transforming-growth factor-beta (TGF-β) expression at the transcriptional level [15] and accelerate TGF-β-induced EMT dramatically [16]. Miettinen et al. first revealed that TGF-β induced EMT in normal mammary epithelial cells [17]. In fact, TGF-β is an important inducer of EMT in cancer progression. In tumor tissues, the interstitial fibroblasts and infiltrating macrophages often produce active TGF-β [18,19]. TGF-β has a dual role in carcinogenesis. In early lesions, TGF-β is considered a major anti-inflammatory cytokine and prevents uncontrolled cell proliferation [20]. However, many advanced tumors are resistant to the growth-inhibitory actions of TGF-β, and TGF-β can instead activate pro-metastatic pathways [21]. While it can act as a tumor suppressor at early tumor stages, TGF-β later contribute to the malignant progression by promoting invasion and metastasis [22]. One mechanism by which TGF-β contributes to cancer progression is the induction of an oncogenic EMT [23]. For instance, TGF-β can directly induce oral squamous cancer cells to a myofibroblastic phenotype, and the TGF-β signaling by stromal myofibroblast can induce secretion of hepatocyte growth factor (HGF) which promotes cancer cell proliferation and invasion [24]. In SMMC-7721 human hepatocellular carcinoma (HCC) cell line, TGF-β could regulate the expression of several integrins, and promoted-EMT and cell adhesion might be both responsible for TGF-β-enhanced cell migration [25]. Among the transcription factors involved in the induction of EMT in cancer, Snail factors repress E-cadherin transcription directly and also activate the transcription of vimentin and α-SMA indirectly [26]. Significantly, TGF-β is also the most potent inducer of Snail transcription. Snail can upregulate the expression of pro-inflammatory mediators (IL-1, IL-6 and IL-8) as well [27]. Thus the relationship between inflammation and EMT seems to be an interaction feature in the progression of cancer (Figure 1).

### Role of Hypoxia and Oxidative Stress in EMT

Based on recent reports, hypoxia may be proposed as a second factor in the initiation of EMT. When tumors grow to a certain size and cancer cells divide uncontrollably, they form larger tumors. As a consequence, there is limited availability of nutrients and oxygen in the microenvironment and cancer cells are exposed to intermittent hypoxic conditions. Higgins et al. have demonstrated that hypoxia-induced EMT in renal epithelial cells depend on hypoxia-inducible factors (HIF) signaling partly [28]. Accordingly, Copple has also suggested that HIF-1α is important for hypoxic to stimulate hepatocyte EMT [29]. Luo et al. used the study with HIF knockdown with siRNA at 2% oxygen and over-expression of an oxygen-insensitive HIF mutant at 21% oxygen to show that HIF regulates Snail activation and subsequent cell migration. The reports identify snail as a HIF target gene and provide novel insights into the regulation of snail and hypoxia-induced EMT [30]. In addition to HIF signaling, cancer cells activate latent TGF-β1 in response to hypoxia. Zhou et al. used the inhibitor of the TGF-β1 type I receptor kinase to prevent the hypoxia-induced EMT, the results suggested that the process was TGF-β1 dependent [31].

During hypoxia, mitochondria increase the production of reactive oxygen species (ROS) and the ROS signaling mechanisms in the cancer cells determine the fate of cancer cells [31](Figure 1). It has been reported that either ROS or nuclear factor kappa B (NF-κB) could facilitate EMT in certain cell types [32-34] and TNF-α could cause NF-κB activation and ROS production [35]. Interestingly, R. Dong pointed out that H_2_O_2 _alone can promote EMT in a way different from TNF-α-induced EMT, in which NF-κB only plays a minor role [14]. Since EMT can be affected by many signal pathways and kinds of transcription factors [36], another transcription factor or signal pathway may be the leading factor of EMT induced by ROS.

### The link between EMT and the cancer stem cells (CSCs) phenotype

The existence of CSCs or tumor initiating cells with the ability to self-renew and give rise to differentiated tumor cells were first reported by Dick and coworkers [37]. Subsequently, the researchers have identified CSCs in several solid tumors originating from the breast, colon and brain [38-40]. Cells undergoing EMT may resist toxic injuries and chemoradiation therapy, and a series of studies demonstrated that CSCs are more resistant to conventional therapies than differentiated cells. Fillmore et al. used the CD44^+^/CD24^-/low^/ESA^+ ^cell surface phenotype to isolate CSCs from human breast cancer cell lines, and demonstrated preferential resistance of CSCs to chemotherapy [41]. Similarly, CD44^+^/CD24^-/low ^cells isolated from monolayer cultures of MCF-7 or MDA-MB-231 cell lines and propagated as mammospheres are also relatively radioresistant, with an increase in the CD44^+^/CD24^-/low ^cell population after irradiation [42]. Therefore, CSCs are considered to be undergoing EMT as well as tumor cells, and EMT may give CSCs the invasive and metastatic abilities necessary for successful metastasis. The chemoradiation-resistant pancreatic cancer cells are rich in ''stem-cell-like'' tumor cells and undergo EMT, the migratory and invasive capacities have been increased in vitro and in vivo [43].

Recent evidence suggests that cells that undergo EMT acquire stem cell-like properties too (Figure 1). Inducing EMT in differentiated HMLE cells by either over expression of Snail or Twist or exposure to TGF-β1 caused the cells to acquire the CD44^high^/CD24^low ^stem cell profile. EMT may also give differentiated tumor cells the ability to self-renew, thus allowing the successful establishment of secondary tumors at distant sites [44]. Furthermore, cancer cells under hypoxic conditions acquire the properties of CSCs. Louie et al. used an optimized hypoxia and reoxygenation regimen to identify a novel cycling hypoxia-selected subpopulation from human breast cancer cell lines. The data demonstrated that a stem-like breast cancer cell subpopulation could be highly tumorigenic in immune-deficient mice and exhibited both stem-like and EMT phenotypes [45].

Another stem cell type that resides predominantly in tumor environment is the mesenchymal stem cells (MSCs), which is a potential candidate of stem cells for cellular and genetic therapy, and can differentiate into multiple lineages such as chondrocytes, osteocytes, adipocytes, myocytes, and astrocytes [46,47]. Recent studies demonstrate that a variety of MSCs from the bone marrow are recruited at injury sites in a number of pathological conditions such as inflammation, tissue repair and also neoplasia [48]. Taking advantage of homing capacities to the primary tumor site, MSCs have been used for the targeted delivery of immunostimulatory cytokines and chemokines, suicide genes, growth-factor antagonists, and oncolytic viruses after systemic administration [49]. However, recent evidence suggests that MSCs participate in tumor growth and metastasis, and are the most prominent cell type within the tumor stroma of many cancers. Subcutaneously implanted human mammary carcinomas co-injected with MSCs acquire an increased metastatic potential [3].

### Complexity of EMT Signaling Pathways in tumor microenvironment

**Figure 2:**
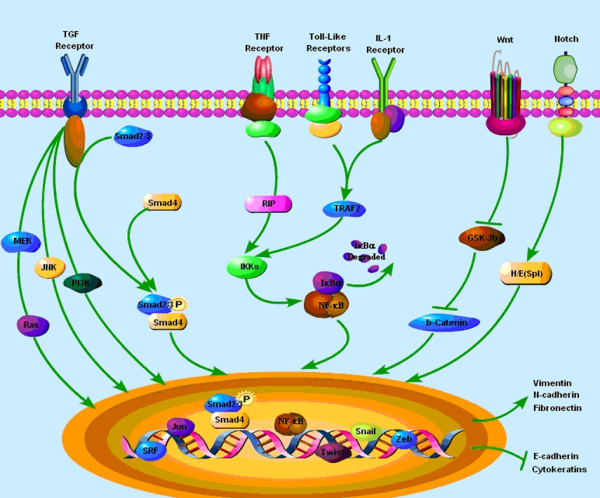
**Signaling pathways that regulate Epithelial-Mesenchymal Transition (EMT) in tumor microenvironment**. EMT is described as a multi-step event that epithelial cells lose numerous epithelial characteristics to assume properties of mesenchymal cells, and the inducers of EMT are complex in tumor microenvironment. Therefore, EMT-related signaling pathways are various, including TGF-β, NF-κB, Wnt, Notch, and others.

EMT is described as a multi-step event that epithelial cells lose numerous epithelial characteristics to assume properties of mesenchymal cells, and the inducers of EMT is complex in tumor microenvironment. Therefore, EMT-related signaling pathways are various, including TGF-β, NF-κB, Wnt, Notch, and others [50] (Figure 2).

TGF-β signaling pathway is a key player in promoting tumor progression and metastasis [22,51]. TGF-β induces tumor EMT through a Smad-dependent transcriptional pathway and a Smad-independent transcriptional pathway [1]. In Smad-dependent pathway, the binding of TGF-β results that TGF-β receptors type I and II form tight complexes leading to phosphorylation of Smad2 and Smad3, the receptor-related Smad (R-Smad) proteins [52]. Phoshorylated Smads then form heteromeric complexes with Smad4 and translocate into the nucleus to control transcription of target genes associated with EMT through interaction with specific binding motifs in their gene regulatory regions, such as Snail, Slug, ZEB and so on [53]. In addition to the Smad signaling pathways, TGF-β directly activates various types of non-Smad signaling in certain types of cells. Among them, it is reported that Ras/Erk, c-Jun N-terminal kinase (JNK), phosphatidylinsitol-3 (PI3) kinase, Par6, and Cdc42 GTPases play important roles in TGF-β-induced EMT [54,55]. Therefore, targeted treatment against TGF-β signaling appears to be promising as high expression of TGF-β is a key mediator of tumor EMT process. For example, TGF-β receptor 1 kinase inhibitor (LY2109761) deactivates Smad-2, decreasing the migration and invasion of HCC cells and up-regulating E-cadherin expression in HCC cell membranes, which mediates cell adhesion [56-58].

In tumor environment, an increase in the expression of the inflammatory cytokines (TNF-α, IL-6, LPS) and ROS under oxidative stress is crucial for the induction of NF-κB pathway, and NF-κB can also directly activate the expression of potent EMT inducers, including Snail and ZEB factors [59]. It has been found that NF-κB suppresses the expression of epithelial specific gene E-cadherin, and induces the expression of the mesenchymal specific gene vimentin. Snail is a central transcription factor during loss of epithelial phenotype to repress E-cadherin expression, and NF-κB has been found to induce the expression of Snail, which leads to the down regulation of E-cadherin [60]. NF-κB also upregulates transcription factor ZEB1 and ZEB2, resulting in the inhibition of E-cadherin expression during EMT [34]. If cells having already undergone EMT, blocking of NF-κB activity leads to a partial reversal of the mesenchymal phenotype [61]. Recent studies identified NF-κB transcription factor as another key modulator of TGF-β-induced EMT. NF-κB can promote EMT in pancreatic carcinoma cells, which are unresponsive to TGF-β since they lack functional SMAD4. Interestingly, TNF-α was still able to elicit an EMT-like phenotype in these TGF-β-unresponsive cells through NF-κB [62]. Therefore, the cooperation of TGF-β and NF-κB is critical for EMT, and plays an important role in cancer invasion and metastasis.

Wnt/β-caternin and Notch pathway are also emerging as important regulators of EMT in carcinoma cell lines, as well as the maintenance of stemness properties of stem cells. Translocation of β-caternin to the nuclear might result in the loss of E-cadherin to induce EMT, and β-caternin signaling is also essential to maintain the stemness properties of CSCs in skin cancer [63]. Transforming growth factor (TGF)-β, canonical and noncanonical Wnt signaling all collaborated to induce activation of the EMT program and thereafter function in an autocrine fashion to maintain the resulting mesenchymal state [64]. Inhibition of Wnt signaling can block EMT transcription factors and promote epithelial differentiation. Recent studies propose Snail2 as a target of Notch signaling, which is one of EMT transcription factors [65]. Blocking the Notch pathway by pharmacologic inhibitors of c-secretase might result in a depletion of CD133 stem-like cells in embryonal brain tumors [66]. Both the two signaling pathways contribute to EMT and to cancer stem-like cell characteristics in tumorigenesis.

## Conclusions

During the past few decades, an increasing number of studies have shown that EMT is associated with cancer progression and metastasis. A variety of factors in tumor microenvironment can lead to EMT. Inflammation, hypoxia, and stem cells in tumor microenvironment linked with EMT inextricably through complex pathways. Current understanding of traditional signal pathways coupled with new concepts in EMT could accelerate progress in cancer research. Furthermore, improved understanding of the tumor microenvironment, which contributes to the maintenance of EMT, could clarify the processes underlying EMT, so as to be targeted. However, a large number of unknown factors and intracellular signaling pathways have been associated with EMT, the multimodal nature of these complex pathways presents will forbid researchers attempting to inhibit the onset of EMT and the clinical significance of challenging the role of EMT in cancer progression is still relatively weak. Thus, better understanding for EMT in tumor microenvironment is still needed.

## List of abbreviations

CSCs: cancer stem cells; ECM: extracellular matrix; EMT: epithelial-mesenchymal transition; HCC: hepatocellular carcinoma; HGF: hepatocyte growth factor; HIF: hypoxia-inducible factors; IL-1: Interleukin-1; MSCs: mesenchymal stem cells; NF-κB: nuclear factor kappa B; ROS: reactive oxygen species; TAMs: tumor associated macrophages; TGF-β: transforming-growth factor-beta; TNF-α: tumor necrosis factor-alpha.

## Competing interests

The authors declare that they have no competing interests.

## Authors' contributions

YY J, ZP H, SS Z and LX W planned the manuscript outline. YY J wrote the draft manuscript, ZP H, SS Z, Y L revised and LX W finalized the manuscript. All authors read and approve the final manuscript.
